# A new method of building permanent A-V block model: ablating his-bundle potential through femoral artery with pre-implanted biventricular pacemaker

**DOI:** 10.1186/1471-2261-14-164

**Published:** 2014-11-20

**Authors:** Zheng Cheng, Ye Hai-ge, Li Jin, Ye Wan-chun, Wang Lu-ping, Li Yue-chun, Lin Jia-Feng

**Affiliations:** Department of Cardiology, Second Affiliated Hospital of Wenzhou Medical University, 109 Xueyuan Road, Wenzhou, Zhejiang China; Department of Hematology, First Affiliated Hospital of Wenzhou Medical University, Wenzhou, 325000 China; Department of TCM, First Affiliated Hospital of Wenzhou Medical University, Wenzhou, 325000 China

**Keywords:** A-V block, His-bundle potential, Biventricular pacemaker, Radiofrequency ablation, Beagle, Disease models

## Abstract

**Background:**

To explore the feasibility of a new method of achieving a permanent A-V block animal model.

**Methods:**

16 beagles were randomly divided into two groups based on the method of their pre-implanted biventricular pacemakers. (1) In the first group (8 beagles), the A-V block model was achieved by ablating his-bundle potential at the site of the left ventricular superior-septum, under the aortic sinus, through femoral artery. (2) In the second group (8 beagles), the A-V block model was achieved by ablating his-bundle potential at the triangle of Koch, through femoral vein. A complete A-V block model was achieved as a standard in this study. The success rates, intraoperative arrhythmias, operative and X-ray exposure time, intraoperative bleeding amount were assessed in this two groups, both animal models were followed up for four weeks and then fasted to monitor myocardial pathological changes.

**Results:**

The success rate of the first group, which with fewer intraoperative arrhythmias, and less operative and X-ray exposure time, was significantly higher than the second group.

**Conclusions:**

Compared with traditional animal method, our new method of ablating his-bundle potential at the left ventricle from the femoral artery has a higher success rate, fewer occurrence of malignant arrhythmias, and less operation and X-ray time. Thus, our new method should be preferred in the building of Permanent A-V Block Model.

## Background

Arrhythmias are usually classified as tachyarrhythmias or bradyarrhythmias. A complete A-V block is a severe kind of bradyarrhythmia, the causes of pathological A-V block are varied and include ischaemia, infarction, fibrosis or drugs. Patients with complete heart block are frequently hemodynamically unstable, and as a result, they may experience hypotension, syncope, cardiovascular collapse, malignant arrhythmias, or even death. The cause of death may often be tachyarrhythmias precipitated by prolongation of ventricular repolarization secondary to the abrupt changes in rate. Unless the heart block is due to a medication that can be discontinued or an infectious process that can be effectively treated, most patients with acquired complete heart block should receive a permanent pacemaker. When treated with permanent pacing, the prognosis is better. So far, the implantation of a pacemaker is generally accepted as a main therapy for this disease in clinic.

To fully understand the pathogenesis of the complete A-V block and to evaluate the therapeutic effects of different interventions for this kind of disease,a convenient, simple, and reliable animal model is needed. Currently, the ablation of his-bundle potential at right ventricular Koch triangles via the femoral vein is widely used for a complete A-V block, but it still has inherent disadvantages such as difficulty in mapping his-bundle potential, no response to his-bundle potential ablation and easily triggered ventricular arrhythmias. Based on the improvements of the traditional method, here we described a new complete A-V block model with less inherent disadvantages, in which we ablated his-bundle potential under the aortic valve in the left superior ventricular septum from the femoral artery to build a complete A-V model.

PREVENT-HF German Substudy has demonstrated a significant advantage of biventricular pacing vs right ventricular pacing for atrioventricular block in terms of spiroergometric exercise capacity in patients without previous advanced heart failure after 1 year [1]. So in our study, we chose biventricular pacing prior to right ventricular pacing, which is different from Gonzalez and Sousa’ experiments.

## Methods

### Animals

A total of sixteen healthy beagles, male, 2-year-old, weight(18.76 ± 1.72)kg, were obtained from Nanjing Yadong Laboratory Animal Research Center [SCXK(S)2011.0013]. All 16 beagles were housed in the laboratory animal center of Wenzhou Medical University. All experiments were carried out in accordance with the China Animal Welfare Legislation and were approved by the Wenzhou Medical University Committee on Ethics in the Care and Use of Laboratory Animals.

### Preoperative preparation

Before operation, beagles were fasted of food for 12 hours and water for 4 hours. The chest, back, and side-limbs’ skin was shaved, washed, and sterilized. Half an hour after intraperitoneal injection of 3% pentobarbital sodium (30 ml/kg) (Xitang Biological), subjects got general anesthesia.

### Implantation of biventricular synchronization pacemaker

After anesthesia, beagles were fixed onto the operation table in the supine position, with back paddle (indifferent electrode) placed over the left scapula. After endotracheal intubation, the beagles were ventilated by means of a respirator (SIMV mode, respiration rate 20 bpm, tidal volumn 10-20 ml/kg, oxygen flow 4-6 L/min, fraction of inspired oxygen 40%-60%) during the operation time, and repeated doses of pentobarbital sodium were infused as necessary throughout the experiment. The Electrocardiograms were continuously recorded throughout the operation. Medtronic CapSureFix Novus(TM)5076 Leads were inserted from subclavian vein, went through the superior vena cava, right atrium, and fixed into the apex of the right ventricle. Three pacing parameters of leads were monitored – the pacing threshold, pacing impedance and R-wave amplitude, for required ventricular pacing threshold (<1.0 V), required R wave amplitude (>5.0 mV), and ventricular pacing impedance in the range from 400 to 1000Ω. The thoracic cavity was opened along the left fifth intercostal space, and the pericardium was opened to fix the Medtronic Capsure Epi4965 leads onto the avascular area of the lateral left ventricular wall before closure of the cavity. A subcutaneous tunnel was made to lay the right ventricle’s endocardial lead from the cervical region to the thoracic region. The endocardial lead and epicardial lead were linked to a biventricular pacemaker, which was put into a pre-made subcutaneous sac on the thoracic region. The function of the pacemaker was tested, and pacing parameters were set with VVI pacing mode and pacing frequency, which had 10 ~ 20 beats every minute more than the subject its own. After confirmation the proper conditions for the pacemaker,we sutured the subcutaneous tissue and skin.

### Ablation of his-bundle potential after implantation of biventricular synchronization pacemaker

After successful implantation of biventricular synchronization pacemakers in the subjects, pacemakers working in VVI mode were established and the VVI pacing frequency was reinstalled to 60 bpm. Next, subjects implanted with biventricular pacemakers were randomly divided into two groups: (1)The first group: Ablation of his-bundle potential in left ventricle (n = 8), weight(18.89 ± 1.56)kg. (2). The second group: Ablation of his-bundle potential in right ventricle(n = 8), weight(18.64 ± 1.40)kg.

In the first group, a 7 F arterial sheath was placed in the femoral artery using Seldinger ’s method, and a 7 F temperature-controlled ablation catheter (Biosence Webster, USA) was delivered to the root of aorta via the arterial sheath in the guidance of X-ray. If the his-bundle potential was found while searching in the non-coronary cusp, we tried to ablate it. If not, the catheter was delivered across aortic valves and into left ventricle. We usually found the biggest his-bundle potential in the area of left ventricular superior septum, near the root of aorta. In addition, if a fixed relationship between A, H, and V potential existed, we tried to ablate it with a temperature of 60°C, energy of 60w, and the impedance varies between 80Ω-150Ω. If the complete A-V block appeared with a consistent VVI pacing rhythm during a 10s discharge, the potential was considered an effective ablation target and received another 60s discharge. If the complete A-V block did not appear in during 10s of ablation, his-bundle potential was reassessed until the effective target potential was found and the A-V conduction was blocked completely. Success (complete AV block) was defined under the criteria that the A-V conduction did not recover, and VVI pacing rhythm consistently existed after close observation of the animals for 30 minutes post-operation. We defined the occurrence of sustained ventricular tachycardia or ventricular fibrillation as endpoint of operation. If these arrhythmias happened, then the operation need to be stopped immediately. We divided the success cases by the total number of attempts to calculate success rate.

The second group received ablation of his-bundle potential in the right ventricle. A 7 F vein sheath was placed in femoral vein using Seldinger ’s method. A 7 F temperature-controlled ablation catheter (Biosence Webster, USA) was delivered to the right ventricle through the vein sheath via X-ray guidance. If a his-bundle potential containing a fixed relationship with A and V potential was found when searching in Koch triangles (in the right ventricular upper septum near tricuspid annulus), discharge was attempted (the temperature, energy and impedance of ablation were the same as those of the left ventricle ablation group, and the ablation success criteria and ablation endpoint were similar to the left ventricle ablation group).

All subjects whom received operations were intravenously injected with 2000 μl of heparin to prevent blood clotting during operation(if the operation time was longer than one hour,additional 500 μl of heparin was added every hour). After ablation, the arterial or venous sheaths were removed, we pressed the points closer to the heart than the sites of puncture to stop bleeding, the blood from the puncture sites were suctioned and collected into a sterile empty vial to calculate the bleeding amount. Operation time was recorded from insertion of sheath to remove of sheaths. All procedures followed principles of aseptic technique. After operation, the biventricular pacemaker pacing frequency was reprogrammed to 130 bpm and the left ventricle pacing was set 10 ms earlier than the right ventricle. Every dog was intramuscularly injected with 1.6 million U/day of penicillin for 3 days to prevent infection. The body surface electrocardiograms (ECG) of subjects were recorded at least once a week.

### Follow-up post-operation

Dogs were followed up with for 4 weeks after operation. Echocardiographs, ECGs, and plasma BNP concentrations were examined at the time of pre-operation, two weeks post-operation, and four weeks post-operation respectively. The plasma BNP level was detected by the quantitative sandwich enzyme immunoassay technique using the BNP elisa kit (MyBioSource MBS922862). Echographs were recorded by ultra-sound machine (Philips CX-30 untra-sound machine). Echocardiograph measurements included:1eft ventricle end diastolic diameter(LVEDD), 1eft ventricle end systolic diameter (LVESD), intraventricular septum diastolic diameter (IVSDD), intraventricular septum systolic diameter (IVSSD), 1eft ventricle ejection fraction (LVEF),fractional shortening (FS) and 1eft atrial internal diameter (LAID), all the datas were measured on parasternal long axis view, the value of LVEF was calculated by M-mode LV dimensional method. ECGs were recorded by ECG machine (Edan SE-1200 express ECG machine). ECGs measurements included: QRS duration, QT interval, Tp-Te interval. Subjects were euthanized four weeks after operation, and the pathological changes of hearts were observed.

### Pathological examination

Ablated myocardial tissue area of the two groups were collected and every specimen were divided into two parts. One part was subjected to formalin fixation, alcoholic dehydration, paraffin embedding, slicing, HE staining, and ultimately used for pathological study. The other part of the tissue was preserved in 4% glutaral, washed with a phosphate buffer, stained with phosphotungstic acid, embedded in Ciba 5o2 with polymerization, cut with a Porter-Blum ultramicrotome, stained with toluidine blue, and ultimately used for ultrastructural study.

### Statistics analysis

Data were presented as Mean ± Standard deviation (x ± s). Independent samples were applied for comparison among groups using the *t*-test and ANOVA analysis. Enumeration data is described as case numbers and percentages. The probability was directly measured among groups through X-squared tests or Fisher’s exact test. Analysis was performed with SPSS 19.0 statistical software. A P value of <0.05 was considered as statistically significant.

## Results

### The building of a complete A-V block model

**Figure 1:**
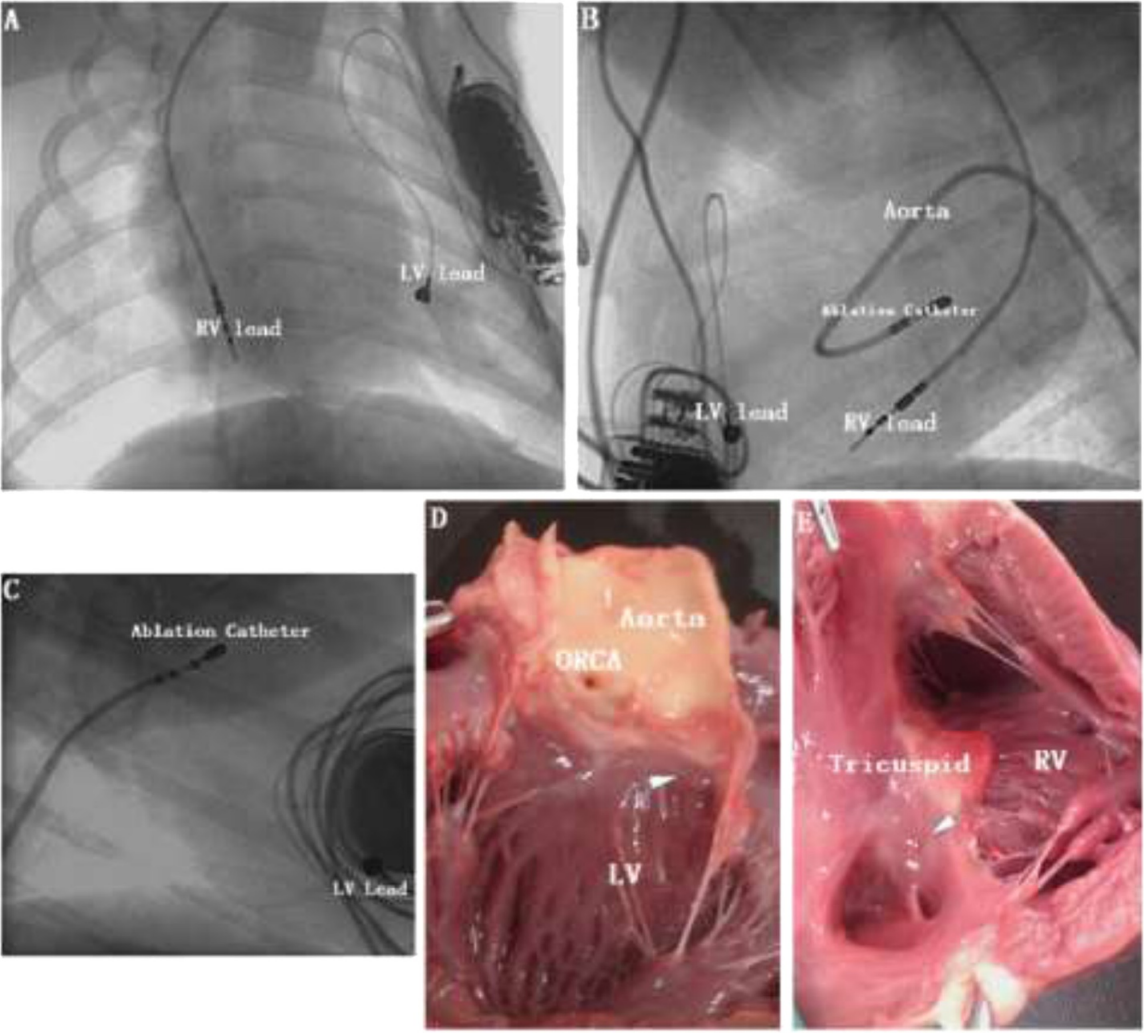
**Pacemaker implantation and His-bundle ablation. A**. Implantation with biventricular pacemaker; **B**. In the position of right anterior oblique (RAO), ablating his-bundle in the left ventricle; **C**. Ablating his-bundle in the right ventricle; **D**. Heart specimen from the first group, the arrow indicates the ablation location; **E**. Heart specimen from the second group, the arrow indicates the ablation location. ORCA: ostium of right coronary artery, RV: right ventricle, LV: left ventricle.

**Figure 2:**
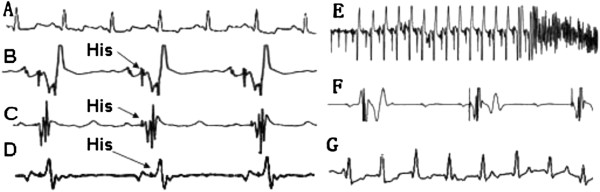
**Electrocardiographs and electrophysiological findings during the proceduces. A**. Electrocardiograph before operation (in lead III, paper speed was 50 mm/sec); **B**. Obvious His-bundle potential at the site of left ventricular upper septum under aortic sinus (paper speed was 150 mm/sec); **C**. His-bundle potential in the right ventricle was much smaller than that in the left ventricle (paper speed was 150 mm/sec); **D**. His-bundle potential in non-coronary sinus (paper speed was 150 mm/sec); **E**. Ventricular fibrillation occurred when ablating his-bundle potential at Koch triangles (inlead III, paper speed was 10 mm/sec); **F**. Right ventricular pacing after ablating his-bundle(leadIII, VVI/60 bpm, paper speed was 50 mm/sec); **G**. Biventricular pacing after operation(lead III, VVI/130 bpm, paper speed was 50 mm/sec).

All the subjects were successfully implanted with pacemakers (Figure 1A). Before the ablation of his-bundle potential, the average heart rate of subjects was 138 ± 9 bpm of sinus rhythm when pacing frequency of pacemaker was installed at 60 bpm. After ablation of his-bundle potential, all pacemakers were in good condition and programmed with a pacing frequency of 130 bpm in VVI mode. Among the 16 subjects, we achieved complete A-V blocks in 14 using the two different ablation methods. The operation time, X-ray exposure time, and amount of bleeding in the two groups were shown in Table 1. (1) In the first group, all the subjects survived after operation. We found an obvious his-bundle potential in the non-coronary cusp in one dog (Figure 2G), but failed to ablate it at this site. The his-bundle potentials in 8 subjects were finally ablated in the area of the left ventricular superior septum under aortic valve near the non-coronary cusp. In this group,we achieved complete A-V block in all subjects without malignant ventricular arrhythmias happened during operation. After ablation of his-bundle potential, subject heart rate was sustained at 130 bpm (VVI pacing rhythm). (2)In the second group, ventricular fibrillation occurred in two subjects when ablating his-bundle at Koch triangles. Despite our best efforts, one subject still died of recurrent ventricular fibrillation, while the other survived but failed to achieve complete A-V block. The rest of the 6 subjects in this group were successfully ablated of his-bundle potential and achieved complete A-V block. The heart rates of the 14 subjects were sustained at 130 bpm (VVI pacing rhythm).

**Table 1:** **Comparison of the two groups during operation**

	Left ventricle ablation group (n = 8)	Right ventricle ablation group (n = 8)
Puncture site	Right femoral artery	Right femoral vein
Ablation site	Left ventricular upper septum under aorta ostium	Right ventricular Koch triangles
Success case	8(100.00%)	6(75.00%)
Operation time (min)	107.55 ± 13.26	151.74 ± 18.32*
X-ray exposure time (min)	10.22 ± 3.67	15.25 ± 3.70*
Amount of bleeding (ml)	33.31 ± 4.30	9.52 ± 1.28*
Cases of malignant ventricular arrhythmias	0	2

Comparison of the right ventricular ablation group with the left ventricular ablation group, *P < 0.05.

### Follow up with electrocardiogram characteristics, echocardiograph parameters, and BNP levels in the two groups

The fourteen subjects (eight from the first group, six from the second group) presented stable VVI pacing rhythm without complications during the 4 weeks follow-up. There were no symptoms or body signs indicating heart failure, such as anorexia, shortness of breath, engorgement of jugular, etc. There were no significant differences in weight, breath, echocardiograph parameter, QT interval, or BNP levels between pre-operation and post-operation in the subjects, except QRS duration and Tp-Te interval(Table 2).

**Table 2:** **The changes of ECG characteristics, ECG parameters and BNP levels in the two groups before and after operation**

	Left ventricle ablation group			Right ventricle ablation		
	Before operation	2 weeks post operation	4 weeks post operation	Before operation	2 weeks post operation	4 weeks post operation
Heart rate (bpm)	140 ± 10	130	130	135 ± 7	130	130
Weight (kg)	18.89 ± 1.56	18.39 ± 1.30	19.19 ± 1.45	18.64 ± 1.40	18.15 ± 1.33	19.27 ± 1.19
Respiration rate (bpm)	23.77 ± 4.93	23.67 ± 4.57	23.19 ± 4.18	22.84 ± 4.85	24.35 ± 4.39	23.65 ± 3.88
QRS duration (ms)	70.51 ± 5.20	77.72 ± 4.17**	80.73 ± 4.25*	70.65 ± 6.50	78.50 ± 5.37**	82.81 ± 6.23*
QT intervals (ms)	202.23 ± 18.42	216.31 ± 17.71	216.10 ± 16.73	201.60 ± 13.25	210.54 ± 13.74	211.18 ± 13.65
Tp-Te interval (ms)	59.52 ± 10.87	70.30 ± 8.44	83.81 ± 9.32*	60.23 ± 12.34	67.15 ± 12.68	83.10 ± 8.06*
LAID (mm)	20.31 ± 1.24	20.49 ± 1.62	20.81 ± 1.45	20.15 ± 1.167	20.81 ± 1.167	20.64 ± 1.26
LVEDD (mm)	18.78 ± 1.85	19.40 ± 1.79	19.88 ± 1.42	18.60 ± 1.85	19.03 ± 1.81	19.51 ± 1.15
LVESD (mm)	22.26 ± 2.50	23.93 ± 0.83	22.42 ± 2.69	21.15 ± 1.70	21.98 ± 1.39	22.65 ± 1.48
IVSDD (mm)	5.64 ± 0.64	5.83 ± 0.74	5.10 ± 0.64	5.61 ± 0.88	5.73 ± 0.17	5.75 ± 0.16
IVSSD (mm)	9.71 ± 0.46	9.91 ± 0.35	10.16 ± 0.53	9.72 ± 0.43	9.85 ± 0.31	9.98 ± 0.45
LVEF (%)	67.01 ± 4.06	63.56 ± 3.82	63.60 ± 1.96	65.98 ± 1.39	63.31 ± 1.19	62.65 ± 1.54
FS (%)	35.15 ± 2.02	35.48 ± 2.49	36.65 ± 1.73	34.81 ± 2.30	34.98 ± 2.59	36.31 ± 1.84
BNP (μg/L)	50.09 ± 15.96	52.70 ± 16.51	54.09 ± 14.66	48.18 ± 16.85	50.10 ± 16.26	51.41 ± 15.50

Comparison of the parameters 4-week post operation with those before operation, *P < 0.05;Comparison of the parameters 2-week post operation with those before operation, **P < 0.05.

LAID: left atrial internal diameter, LVEDD: left ventricle end diastolic diameter, LVESD: left ventricle end systolic diameter, IVSDD: intraventricular septum diastolic diameter, IVSSD: intraventricular septum systolic diameter, LVEF: Left ventricle ejection fraction, FS = fractional shortening.

### Pathological examination

**Figure 3:**
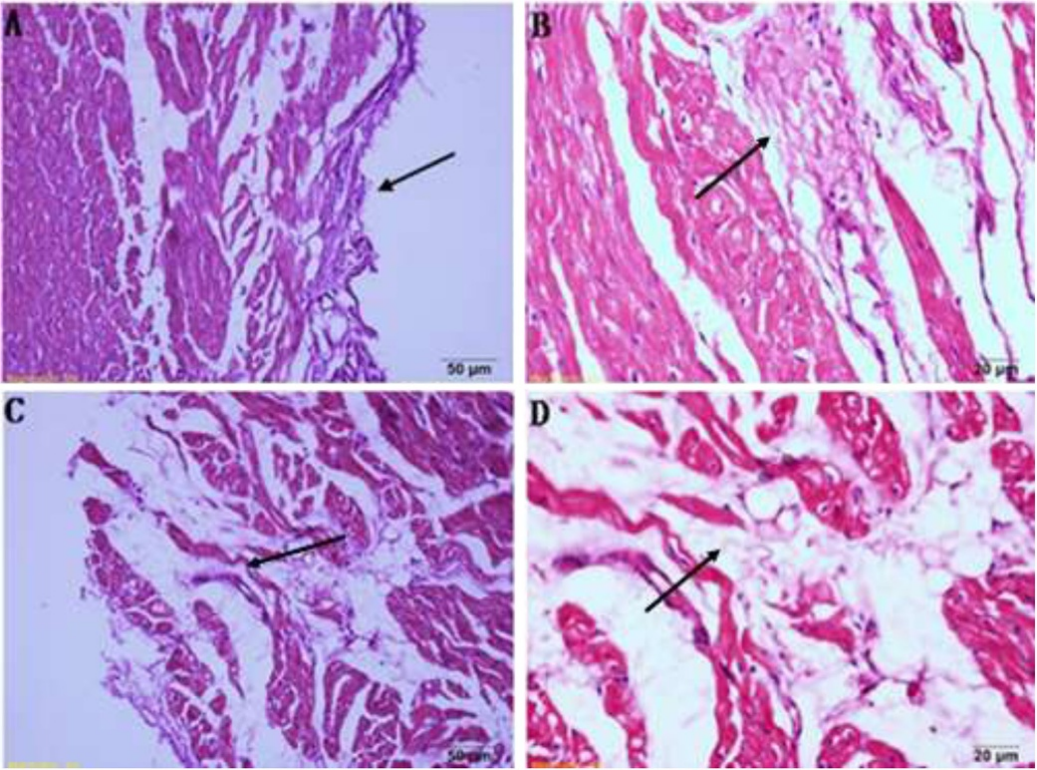
**HE staining of ablated tissue. A**. Light microscope of the first group (×20), ablated tissue presented as coagulation necrosis. **B**. Light microscope of the first group (×40), proliferation of the fibroblast cells. **C**. Light microscope of the second group (×20), infiltration of chronic inflammatory cells. **D**. Light microscope of the second group (×40), vacuolization of lipocytes.

**Figure 4:**
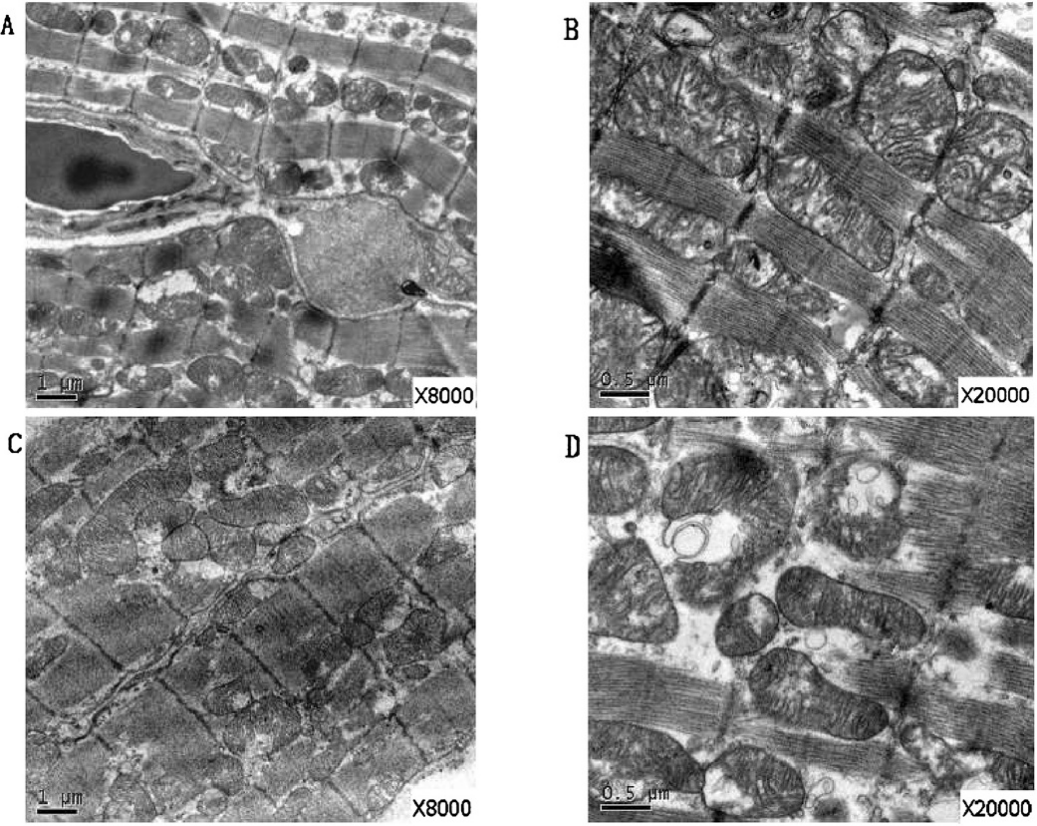
**Electron microscope of ablated tissue. A**. Electron microscope of the first group (×8000), aligned myofibrils were full of the cytoplasm, the structure of a sarcomere kept integral; **B**. Electron microscope of the first group (×20000), abundant mitochondria located between nuclei and myofibrils, and contained intensive cristae full of inner mitochondrial space; **C**. Electron microscope of the second group (×8000), the myofibriles and mitochondria got swollen; **D**. Electron microscope of the second group (×20000), mitochondria got irregular and the inner cristae became sparse.

Under the light microscope, the post ablation tissue showed elliptical coagulation necrosis, which could be easily distinguished from normal tissue. Myocardial fibrosis, infiltration of lipocytes, and chronic inflammation of cells could be clearly seen in the tissue of ablated (Figure 3). As shown by electron microscope, the ultrastructure of ablated tissue had changed markedly. Under 8000x-magnification (Figure 4A, C), a longitudinal section of the cytoplasm was full of aligned myofibrils, I zone, A zone, H zone, and Z line of myofibrils, making up the structure of a sarcomere. The cytoplasm was filled with abundant mitochondria, most of which were round or oval in shape, located between nuclei and myofibrils, and contained intensive cristae full of inner mitochondrial space. Under 20000x magnification, many mitochondria contained incomplete externa and were swollen, showing reduction and sparseness of cristae (Figure 4B,D). Additionally, some mitochondria vacuolized and disappeared.

## Discussion

In 1981,Gonzalez reported ablation of his-bundle potential at Koch triangles through the femoral vein and complete atrioventricular (AV) block was induced in 9 of 10 dogs [2]. After that, we followed this method to build a complete A-V block animal model. In our research, we proposed a method of build complete A-V block animal model - ablation of his-bundle potential through the left ventricle, meanwhile, we have demostrated that our method has higher success rates (100% vs 75%), less occurrence of malignant arrhythmias(0 cases vs 2 cases), shorter operation time (107.55 ± 13.26 min vs 151.74 ± 18.32 min, P < 0.05) and X-ray exposure time(10.22 ± 3.67 min vs 15.25 ± 3.70 min,P < 0.05) compared with traditional method of ablation His-bundle through right ventricle.

### The basis of ablation His-bundle through left ventricle

Anatomically the common stem of the bundle of His is composed of a non-branching portion and a branching portion. The former is further divided into penetrating and non-penetrating parts. The penetrating part of non-branching portion is enclosed by central fibrous body. The non-branching portion of the common stem passes through the right fibrous trigon to reach the top of the interventricular septum along the posterior and inferior margin of the membranous portion where it begins to bifurcate. From this anatomic standpoint, it is conceivable that the his-bundle potential can be recorded when the catheter electrodes are positioned adjacent to the membranous septum (right below the aortic valve and between the noncoronary cusp and right coronary cusp).In 1975, Ying-shiung reported recorded the electrical potential of the His-bundle potential from left ventricular endocardial surface in 28 patients [3]. In their presentation, they utilized a bipolar electrode catheter placed in the subaortic region (with the tip directed medially and slightly posteriorly) to record His-bundle potential. In 1981, Joao Sousa demostrated the feasibility of ablating AV conduction using radiofrequency energy delivered in the left ventricle in arrhythmias patients [4]. The technique was successful in eight patients with refractory supraventricular tachycardia in whom a conventional approach with a catheter across the tricuspid valve had failed to produce AV block. The His bundle potentials recorded in the left ventricle were generally larger than those recorded in the standard position across the tricuspid annulus. There were no complications related to the procedure. In 1982, Olga souza reported in six patients in whom a right-sided of ablation of AV conduction approach was initially used,but failed [5]. He thought although the His bundle was correctly mapped from the electrical point of view, contact of the electrode may have been less than satisfactory. However, a left-sided approach was used in these patients, resulting in block in AV conduction. Fewer applications of RF energy were required from the left side to obtain complete AV block. He concluded that catheter ablation of His bundle is easier using a left-sided approach than using a right-sided approach, probably because of a firmer tissue contact. The left-sided approach reduces procedure and radiation time avoiding complications and recovery of conduction and may be preferable to conventional right-sided approach.

All these reserches are performed on patients and accorded with our study on animal. We firstly demostrated that in animal model, ablation his bundle from left sided way is prior to right side. Ablation his bundle from left sided way has higher success rates. Furthermore, our method exhibited a less operation and X-ray exposure time, as well as less malignant arrhythmias.

### Biventricular pacing and transmural dispersion repolarization

In our model, successful ablation of His bundle resulted in a long dependency on biventricular pacemker. We followed the ECG of the subjects and found that after 4 weeks of pacing relying on biventricular pacemaker, the QRS duration and Tp-Te interval were apparently increased compared with preoperation. The Tp-Te interval provides a measure of the transmural dispersion of repolarization(TDR) [6–10]. Recent studies show a prolong Tp-Te has been linked to spontaneous development of ventricular tachycardia [11]. It is well established that cardiac resynchronisation therapy (CRT) using biventricular pacing prolongs survival by its effect on pump failure [12–15]. Several studies have suggested that CRT suppresss the incidence of major arrhythmic events, citing reduced wall stress and decreased repolarization dispersion (as a result of dual depolarization wavefronts) as potential mechanism [16–18]. Other studies, however, have demostrated a proarrhythmic potential [6, 19–22]. Recent reports suggest that left ventricle epicardial pacing can be proarrhythmic, leading to polymorphic ventricular tachycardia (VT) by reversal of the normal activation sequence, prolongation of transmural dispersion of repolarization. LV epicardial pacing reverses the natural activation sequence from endocardium to epicardium. This reduces the repolarization time of the already short epicardial action potentials, thereby increasing repolarization time differences compared with the longer underlying action potentials of the midmyocardial and endocardial layers. Thus, TDR may contribute to ventricular arrhythmias. In summary, the relationship between the TDR and biventricular pacing is remain a question. In our study,there was a significant difference in the Tp-Te intervals of electrocardiograms between post-operation and pre-operation which supported the perspective that biventricular pacing increased the transmural dispersion repolarization.

### Disadvantages of our method

Though our method is prior to right-sided method in many aspects, it still has its inherent disadvantages. Ablation his-bundle from left-sided need the puncture of femoral artery. In general, an arterial puncture is an innocuous procedure, but occasionally complications may occur, such as bleeding, aneurysm, A.V. fistula, embolism and so on. We found that femoral arterial puncture resulted in larger bleeding amount than the femoral venous puncture in our study (33.31 ± 4.30 vs 9.52 ± 1.28, P < 0.05). However, awareness of the complications of arterial puncture may help us to avoid their occurrence.

### Limitation

In our study, we found that in dogs, ablation his bundle in the right ventricle was easier triggering malignant arrhythmias than in the left ventricle. However, so far we just only reported this phenomenon, but could not explain it. We think it need some further electrophysiological studies. And we will endeavor to explain it in the future.

## Conclusions

Our study shows that the ablation of his-bundle potential under the aortic sinus of the left ventricular upper septum is better than the ablation at the right ventricular Koch triangles in the generation of A-V block model. The new A-V block model is a reproducible model for the study of cardiac function.
